# Rehabilitation Protocols for Surgically Treated Acetabular Fractures in Older Adults: Current Practices and Outcomes

**DOI:** 10.3390/jcm14144912

**Published:** 2025-07-10

**Authors:** Silviya Ivanova, Ondrej Prochazka, Peter V. Giannoudis, Theodoros Tosounidis, Moritz Tannast, Johannes D. Bastian

**Affiliations:** 1 Department of Orthopedic Surgery and Traumatology, Inselspital, Bern University Hospital, 3010 Bern, Switzerland; 2 Department of Plastic and Hand Surgery, Inselspital, Bern University Hospital, 3010 Bern, Switzerland; 3 Academic Department of Trauma & Orthopedics, School of Medicine, University of Leeds, Leeds LS2 9JT, UK; 4 Department of Orthopedic Surgery, Medical School, University of Crete, 71110 Heraklion, Greece

**Keywords:** rehabilitation, acetabulum, surgery, older patients, THA, CHP

## Abstract

**Background/Objectives**: Acetabular fractures in older adults pose significant challenges due to bone fragility, complex fracture patterns, and increased comorbidities. Surgical management, including isolated open reduction and internal fixation (ORIF) and ORIF combined with acute total hip arthroplasty (THA) (combined hip procedure—CHP), have advanced considerably. Nevertheless, optimal postoperative rehabilitation and particularly weight-bearing (WB) recommendations remain controversial and inconsistent. This review aims to assess rehabilitation protocols, focusing on WB strategies following the surgical treatment of acetabular fractures in older adults. It also examines differences in WB restrictions by surgical technique (ORIF vs. CHP) and their impact on recovery, complications, reoperations, and mortality. **Methods**: A systematic review of PubMed, Embase, and the Cochrane Library (2006–2024) included studies involving patients aged ≥65 years treated surgically for displaced acetabular fractures. Data included WB protocols (full, partial, toe-touch), length of stay (LOS), healing, functional outcomes (mobility, Harris and Oxford Hip Scores), complications, reoperations, delayed THA, compliance, readmission, and mortality. Due to heterogeneity, findings were narratively synthesized. Risk of bias was assessed using ROBINS-I and RoB2. **Results**: Twenty studies involving 929 patients (530 isolated ORIF, 399 CHP) were analyzed. The overall mean follow-up was 3.5 years (range: 1–5.25 years). Postoperative WB protocols were reported in 19 studies (95%). Immediate full WB was permitted in 0% of isolated ORIF studies (0/13), with partial WB recommended by 62% (8/13) for durations typically between 6 and 12 weeks. On the other hand, immediate full WB was allowed in 53% (9/17) of CHP studies. Functional outcomes were moderate following isolated ORIF (mean HHS: 63–82 points), with delayed THA conversion rates ranging from 16.5% to 45%. CHP demonstrated superior functional outcomes (mean HHS: 70–92 points), earlier independent ambulation, and higher patient satisfaction (74–90%), yet increased orthopedic complications, including dislocations (8–11%) and implant loosening (up to 18%). LOS varied from 12 to 21 days (mean 16 days) for isolated ORIF and from 8 to 25 days (mean 17 days) for CHP. Readmission within 30 days was not explicitly reported in any study. Mortality at 1 year varied significantly (ORIF: 0–25%; CHP: 0–14%), increasing markedly at long-term follow-up (up to 42% ORIF, up to 70% CHP at five years). Compliance with WB restrictions was monitored in only two studies (11%). **Conclusions**: Postoperative rehabilitation after acetabular fracture surgery in older adults remains inconsistent and lacks standardization. Combining ORIF with acute THA may enable earlier weight-bearing and improved short-term function but carries risks such as dislocation and implant loosening. In contrast, isolated ORIF avoids these implant-related complications but often requires prolonged weight-bearing restrictions. Robust evidence is still missing. Future trials are essential to establish standardized protocols that balance mechanical protection and functional recovery.

## 1. Introduction

Acetabular fractures in older adults represent a significant clinical challenge due to the complexity of fracture patterns, bone fragility, and the presence of multiple comorbidities [1,2]. The incidence of these fractures has been steadily increasing because of the increasing longevity of the aging population with osteoporosis and the reported higher frequency of low-energy falls [3,4]. Consequently, surgical management has become more frequent and increasingly involves complex procedures, reflecting advancements in operative techniques as well as higher expectations regarding functional outcomes and quality of life in this vulnerable population [5].

Surgical intervention primarily consists of two distinct approaches: joint reconstruction-preserving procedures, such as open reduction and internal fixation (ORIF), and joint-replacing strategies, typically ORIF combined with acute total hip arthroplasty (THA) [6,7,8,9]. The main objective of ORIF is the anatomical reduction of the articular surface, which is a critical predictor of clinical success, minimizing the risk of post-traumatic osteoarthritis and subsequent need for conversion to delayed THA [10,11,12]. Indeed, previous studies have identified non-anatomical fracture reduction and dome impaction as significant independent predictors of poor clinical outcomes and increased likelihood for conversion surgery [10,13,14,15]. Conversely, ORIF combined with acute THA (CHP, “fix-and-replace”) provides immediate joint stability, especially in cases with pre-existing osteoarthritis, severe comminution, or anticipated inability to achieve an anatomical reduction, potentially reducing the risk of delayed conversion to THA, albeit at the expense of increased risks of other orthopedic complications and revision surgery [7,16].

Significant variability in postoperative rehabilitation protocols is evident after the surgical management of these injuries, particularly concerning the weight-bearing recommendations, mainly due to ongoing uncertainties about fracture fixation stability, implant integrity, and patient compliance to postoperative limitations [17]. Recent systematic analyses underscore substantial inconsistencies pertaining to optimal timing and type of weight-bearing, whilst at the same time, traditional restrictive protocols are being questioned [5]. Despite the recognized importance of early mobilization in rehabilitation, concerns regarding mechanical stability, fracture fixation integrity, and implant durability frequently result in surgeons recommending conservative postoperative weight-bearing restrictions [17]. The ongoing controversy around optimal timing and degree of weight-bearing reflects a significant knowledge gap, particularly regarding the balance between minimizing immobilization-related complications and protecting surgical fixation in older adults [5].

Consequently, the aim of this systematic review is to evaluate the existing literature on postoperative rehabilitation protocols following surgical management of displaced acetabular fractures in older adults. Specifically, this review seeks to address the following questions:

1. Do reported rehabilitation protocols differ according to the type and timing of postoperative weight-bearing (WB) restrictions?
2. Do surgeons typically restrict postoperative weight-bearing to protect fixation integrity after isolated ORIF?
3. Is the decision to perform a “fix-and-replace” procedure primarily driven by the aim of permitting immediate full weight-bearing postoperatively, or is it predominantly based on established indications, such as pre-existing symptomatic osteoarthritis, severe comminution, femoral head damage, advanced osteoporosis, or the inability to achieve an anatomical reconstruction of the articular surface?
4. Is unrestricted postoperative weight-bearing correlated with shorter hospitalization times, faster healing, superior functional outcomes, lower systemic complications, and decreased mortality, or does restricted postoperative weight-bearing result in fewer orthopedic complications and lower reoperation rates?

## 2. Materials and Methods

### 2.1. Search Strategy and Selection Criteria

This systematic review was conducted according the PRISMA guidelines to ensure a systematic and transparent approach to identifying and analyzing the existing literature [18]. The primary objective was to analyze the postoperative rehabilitation protocols for older adults (≥65 years) after surgical treatment for displaced acetabular fractures, with a focus on weight-bearing recommendations and their clinical implications.

A comprehensive literature search in PubMed/MEDLINE, Embase, and the Cochrane Library was performed to identify relevant studies published between 1 January 2006 and 31 December 2024. The search strategy utilized multiple synonyms and variations for each key term in “all fields”, including “acetabul*”, “geriatric”, “operat*”, and “limit*”, encompassing various spellings and word forms. The search was supplemented by including various synonyms to the aforementioned search terms, e.g. search term “geriatric” and the synonyms “elder*”, “senior”, “older”. The full search terms and search strategy with synonyms can be viewed in the Supplementary Materials (Table S1). Only full-text publications available in English or German were included. We included observational studies, randomized controlled trials (RCTs), multicenter studies, evaluation studies, and pragmatic clinical trials. Exclusion criteria included studies reporting on non-surgical treatment approaches and periprosthetic fractures, case reports, expert opinions, and review articles.

### 2.2. Study Selection and Data Extraction

We used EndNote 20 (Clarivate Analytics, Philadelphia, PA, USA) to analyze the collected data. After removing duplicates, two independent reviewers (SI and OP) evaluated titles and abstracts based on predefined criteria to determine eligibility, and a thorough full-text evaluation of the potentially eligible studies was performed. Any discrepancies were resolved by a third reviewer (J.D.B.) to reach consensus.

To ensure consistency and completeness, a standardized data extraction form was utilized to systematically collect essential study characteristics, including study design; patient demographics (age, comorbidities); fracture classification; surgical interventions (ORIF alone, and ORIF combined with acute THA); postoperative weight-bearing protocols (full, weight-bearing as tolerated, partial, touch-toe) and their duration; patient compliance with rehabilitation protocols (measured by adherence to prescribed weight-bearing and physiotherapy guidelines, if reported); duration of follow-up; functional outcomes (mobility scores, implant survival, revision rates until final follow-up); and incidence of complications and mortality. Extracted data were subsequently used for data synthesis and analysis.

### 2.3. Risk of Bias and Level of Evidence

Risk of bias assessment was conducted using ROBINS-I [19] for non-randomized studies and RoB2 [20] for randomized controlled trials. For the ROBINS-I tool, seven domains were analyzed: (1) bias due to confounding; (2) bias in selection of participants into the study; (3) bias in classification of interventions; (4) bias due to deviations from intended interventions; (5) bias due to missing data; (6) risk of bias in measurement of outcomes; and (7) risk of bias in selection of the reported result. An overall assessment was then derived from these domains. For randomized studies, RoB2 included five domains: (1) risk of bias arising from the randomization process; (2) risk of bias due to deviations from intended interventions; (3) risk of bias due to missing outcome data; (4) risk of bias in measurement of the outcome; and (5) risk of bias in selection of the reported result. An overall risk-of-bias judgment was likewise determined based on these domains. The level of evidence was utilized to assess the quality of the studies, following the guidelines of “The Oxford Level of Evidence” [21].

### 2.4. Statistical Analysis

Extracted data were synthesized narratively to describe and compare weight-bearing recommendations across different surgical techniques. Descriptive statistics were applied where appropriate to highlight trends in rehabilitation practices, the extent of adherence to weight-bearing protocols, and associated patient outcomes. Although a full quantitative meta-analysis was not feasible due to variability in postoperative rehabilitation strategies, an exploratory random-effects meta-analysis was conducted for delayed THA conversion rates following isolated ORIF, as this outcome was consistently reported across studies.

## 3. Results

### 3.1. Study Selection

**Figure 1:**
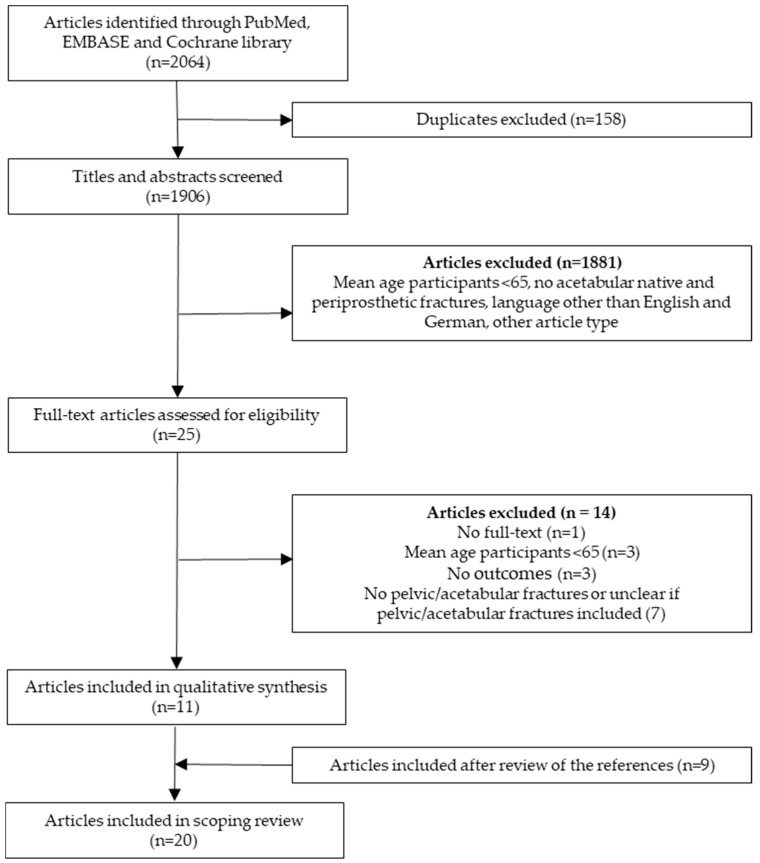
PRISMA flow diagram showing the systematic review protocol.

A total of 2064 articles were initially retrieved, and 158 duplicates were removed (Figure 1). After screening the titles and abstracts, 1881 studies were excluded, leaving 25 full-text articles for assessment. Out of these, 14 were not fulfilling the inclusion criteria and were consequently excluded, thus resulting in 11 articles for the final analysis. Furthermore, reference screening of these included studies yielded nine relevant articles that met the predetermined eligibility criteria. Accordingly, the final systematic review comprised 20 articles. A detailed description of all included studies is presented in Table 1.

### 3.2. Risk of Bias and Level of Evidence

**Figure 2:**
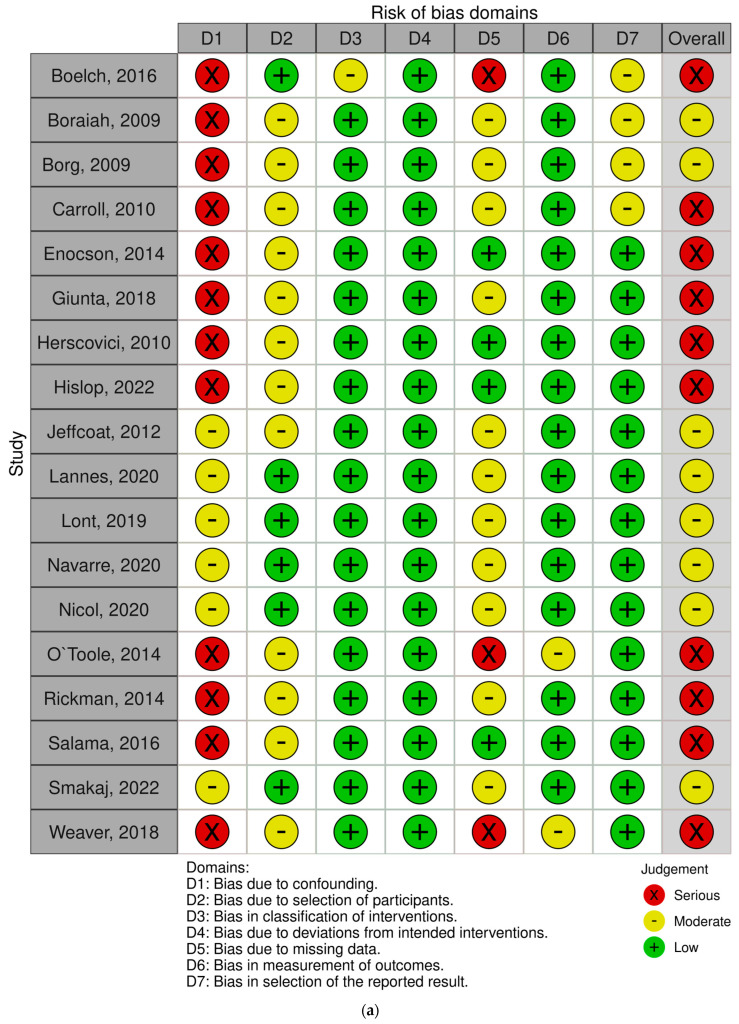

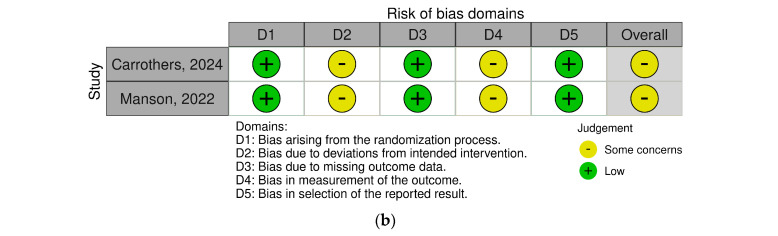
(**a**) Risk of bias assessment (ROBINS-I) of included non-randomized studies [22,23,24,25,27,28,29,30,31,32,33,35,36,37,38,39,40,41]. (**b**) Risk of bias assessment (ROB2) of included randomized studies [26,34].

The overall risk of bias was assessed using the ROBINS-I tool for the 18 [23,24,25,27,28,29,30,31,32,34,35,36,37,38,39,40,41,42] included non-randomized studies and the RoB 2 tool for the two randomized controlled trials (RCTs) [26,34]. Of the 18 non-randomized studies, none were classified as having a low overall risk of bias, eight were rated as moderate risk [23,30,31,32,35,36,40,42], and ten demonstrated a serious risk [24,25,27,28,29,34,37,38,39,41]. The primary methodological limitations among these ten studies included biases arising predominantly from confounding factors, such as insufficient control or adjustment for baseline differences between groups, inadequate reporting of follow-up data, and inconsistent selection criteria. In addition to confounding, serious issues related to missing data were frequently encountered across studies, often due to inadequate reporting of follow-up periods or the lack of explicit handling of participant dropouts. Biases related to participant selection were also prevalent, reflecting inadequate or inconsistent inclusion and exclusion criteria across studies (Figure 2a). Explicit subgroup analysis according to surgical categories revealed no clear systematic pattern in the distribution of serious bias risk: among studies directly comparing both techniques (ORIF and combined ORIF and acute THA), six were rated as moderate risk [23,30,31,32,35,42] and four as serious risk [34,36,38,41]; among studies exclusively evaluating combined ORIF and THA, six were rated as serious [24,25,27,28,29,39] and none as moderate; among studies exclusively evaluating ORIF, one was rated serious [37] and one moderate [40].

Both included RCTs were assessed as having “some concerns” primarily driven by potential deviations from the intended interventions, such as incomplete adherence to study protocols or unclear descriptions of co-interventions [22,33]. Additionally, outcome measurement and reporting issues contributed to this rating, reflecting possible biases arising from unblinded outcome assessors or incomplete reporting of outcome measures (Figure 2b).

### 3.3. Characteristics of the Studies and Demographics

This review included a total of 929 patients from 20 studies published between 2009 and 2024. Of these, 530 patients underwent ORIF alone, and 399 patients were treated using a CHP. The mean age of all patients across studies was 73 years (range: 66–81 years). Patients treated with ORIF alone had a mean age of 71 ± 3 years (range: 67–76 years). Patients who underwent CHP had a higher mean age of 75 ± 4 years (range: 66–80 years). The Judet and Letournel classification was used to classify the fractures in all studies [42]. The most common fracture types across all treatments were both-columns fractures, anterior column posterior hemitransverse (ACPHT), isolated posterior wall fractures, and transverse-posterior wall (Table 1). Notably, certain fracture characteristics were associated more commonly with patients receiving CHP. These included significant femoral head involvement, severe dome or marginal impaction, extensive posterior wall comminution, and substantial fracture complexity with fracture fragments or extensive intra-articular displacement (Table 1). When analyzing the indications for performing CHP versus ORIF alone, 14 of the 20 included studies (70%) clearly based their surgical indication on fracture-specific factors, such as severe joint comminution, femoral head injury, substantial acetabular impaction, or pre-existing symptomatic osteoarthritis [22,24,26,27,28,29,30,32,33,34,35,36,37,40]. Among these, four studies (20%) cited the advantage of enabling immediate or early full weight-bearing and earlier functional rehabilitation as additional decisive factors influencing their choice of CHP over isolated ORIF [22,24,33,40]. Additionally, four studies mentioned other patient-related factors influencing their decision to perform CHP, including the risk of fixation failure [24], patient cognitive impairment and medical comorbidities [26], advanced patient age [28], and medical comorbidities [35]. Notably, six studies (30%) did not specifically report any of these indications clearly [23,25,31,38,39,41].

Of the 20 studies analyzed, two studies (10%) exclusively utilized open reduction and internal fixation without performing any form of acute total hip arthroplasty [31,37]. Ten studies (50%) either exclusively reported the combination of ORIF with simultaneous acute THA [23,27,28,29,30,39] or directly compared ORIF alone with ORIF combined with acute THA [24,26,32,40]. The remaining eight studies (40%) described a treatment strategy that included ORIF alone, ORIF combined with acute THA, and directly identified delayed THA as a possible revision procedure following ORIF [22,25,33,34,35,36,38,41]. As delayed THA was consistently reported as a revision measure due to complications such as secondary post-traumatic osteoarthritis, persistent or worsening joint pain, symptomatic deterioration, failure of initial fracture fixation, progressive articular surface damage, or significant functional impairment after primary ORIF, these studies remained classified within the ORIF group. A detailed breakdown of the surgical distributions is presented in Table 1. The overall mean follow-up across all studies was approximately 3.5 years, with a range from a minimum of 1 year [26,34,40] to a maximum of 5 years [25,31] (Table 1).

### 3.4. Comorbidities and Associated Injuries

Out of the 20 reviewed studies, 17 studies (85%) clearly documented patient comorbidities, whereas three studies (15% [34,39,41]) provided no specific data regarding comorbid conditions. The documentation of comorbidities involved various standardized indices and qualitative assessments. The American Society of Anesthesiologists (ASA) Score was reported in eight studies (40%) [22,24,27,28,30,38,40,41], typically ranging between ASA II and III, indicative of patients with mild to moderate systemic disease. Notably, higher ASA scores (III–IV) were particularly evident in patient cohorts undergoing combined hip procedures (CHP) [38,41].

The Charlson Comorbidity Index (CCI) was directly mentioned in five studies (29%) [30,32,33,35,36]. The reported scores ranged from relatively low comorbidity prevalence (68.1% with CCI 0 [35]) to significantly higher comorbidity levels (mean CCI ≥4 [30,33,36]).

Four additional studies (23%) provided explicit qualitative descriptions of comorbidities without using numerical indices [23,26,29,31]. These descriptions typically included cardiovascular disease, hypertension, diabetes mellitus, pulmonary disease, osteoporosis, neurological disorders, and osteoarthritis.

Furthermore, 13 studies (65%) documented associated injuries alongside acetabular fractures [23,24,25,26,27,28,29,30,31,33,34,35,40,41]. These additional injuries frequently included concomitant orthopedic trauma such as long-bone fractures and hip dislocations, as well as complex high-energy trauma scenarios involving motor vehicle collisions, pedestrian accidents, and significant falls [23,24,41]. Injuries involving the head, thorax, abdomen, and spine were also commonly reported [23,24,25,26,27,28,29,30,31,33,34,35,40,41].

Regarding cognitive status, eight studies (40%) reported the presence of dementia or cognitive impairment among patients [23,24,26,29,30,36,38,41]. The prevalence of dementia or cognitive impairment varied notably among these studies. Specifically, dementia identified either preoperatively or postoperatively was reported in approximately 5.5% of patients by Boraiah et al. [23], 4.5% by Herscovici et al. [29], and approximately 5–10% by Carrothers et al. [26]; Nicol et al. [36] reported the highest explicit proportion of dementia cases at 33.3% (four patients). Hislop et al. [30] identified postoperative transient cognitive dysfunction, not classified directly as dementia, in 15.7% of patients. Borg et al. [24] noted dementia cases in both ORIF and CHP groups without reporting specific percentages, while Rickman et al. [38] and Weaver et al. [41] qualitatively mentioned dementia without detailed numerical data. Moreover, three studies (15%) excluded patients with dementia or significant cognitive impairment as part of their inclusion criteria [27,28,34].

### 3.5. Weight-Bearing Protocols and Compliance

This review revealed considerable heterogeneity regarding postoperative weight-bearing (WB) protocols. Overall, 19 of the 20 included studies (95%) that clearly reported WB restrictions [22,24,25,26,27,28,29,30,31,32,33,34,35,36,37,38,39,40,41]. When analyzing these 19 studies, some studies included both treatment groups, thus leading to subgroup overlap. Specifically, 13 of 14 studies (93%) evaluating isolated ORIF documented WB protocols [22,25,26,27,29,30,32,34,35,36,37,38,40,41], and 17 of 18 studies (94%) evaluating combined ORIF and acute THA clearly reported WB restrictions [22,24,25,26,27,28,29,30,31,32,33,34,35,36,37,39,41]. However, clear documentation and specific recommendations varied notably depending on the surgical treatment approach (Table 2).

For isolated ORIF, partial WB was most frequently recommended (62%, 8/13 studies), with typical durations ranging from approximately 6 to 8 weeks [26,27,33,36,40] up to about 12 weeks [23,29,37,38,41]. Touch-toe or touch-down WB was the second most common protocol (38%, 5/13 studies) [26,31,34,37,41]. Notably, immediate full WB was never permitted following isolated ORIF in any of these studies. One additional study [23] did not explicitly specify the exact WB protocols within the isolated ORIF group. Terms such as ‘touch-toe’, ‘touch-down’, or ‘partial weight-bearing’ were inconsistently used across studies, with no uniform or standardized definitions provided. Most studies did not specifically quantify the permitted weight-bearing in kilograms or as a percentage of bodyweight, leaving these recommendations ambiguous. Only two studies explicitly defined partial weight-bearing with a quantitative limit of 20 kg [32,33].

**Figure 3:**
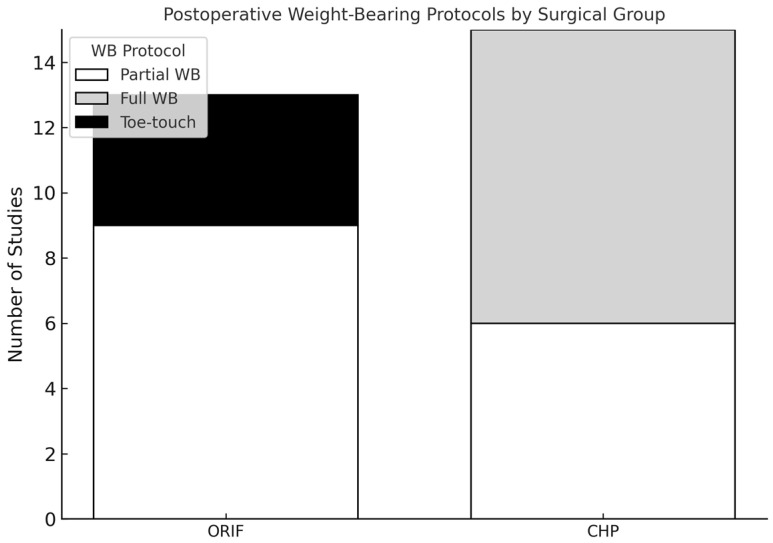
Distribution of postoperative weight-bearing protocols by surgical treatment strategy. The figure summarizes reported postoperative weight-bearing (WB) recommendations across studies, stratified by surgical treatment. ORIF = open reduction and internal fixation; CHP = combined hip procedure (ORIF with acute total hip arthroplasty).

In contrast, for combined ORIF and acute THA, immediate full WB was permitted in approximately half of the studies (53%, 9/17 studies) [26,27,28,30,32,35,36,38,40] (Figure 3). Partial WB was recommended in fewer studies (29%, 5/17 studies) [22,29,33,34,41], typically for durations between 6 and 12 weeks. Touch-toe or touch-down WB was least frequently reported (18%, 3/17 studies) [23,28,39].

The reasons or explicit decision-making criteria behind selecting specific WB protocols were rarely documented across studies. When reasons were provided, they predominantly focused on concerns related to mechanical stability, fracture fixation integrity, and the necessity to protect implants from premature loading. However, no standardized or consistent criteria for determining WB limitations were clearly identified throughout the literature.

Explicit compliance monitoring was performed in only two studies (11%, 2/19 studies): Carrothers et al. [26] conducted regular clinical and radiographic follow-up assessments, while Herscovici et al. [29] indirectly monitored compliance through physiotherapist evaluations. However, in the majority of studies (89%, 17/19 studies), patient adherence to prescribed WB protocols remained entirely unclear.

### 3.6. Duration of Hospitalization and Time to Fracture Healing

Duration of hospitalization (length of stay, LOS) was reported in 10 of the 20 reviewed studies (50%) [22,24,29,30,32,33,37,38,39,40]. For isolated ORIF, LOS averaged 16 ± 3 days (range 12–21), and for combined ORIF and acute THA, LOS averaged 17 ± 5 days (range 8–25 days).

Healing durations varied widely across studies. Fractures treated with isolated ORIF were reported to heal by final follow-up (9 months to 2 years) [24,25,26,35], and those treated with combined ORIF and acute THA from approximately 3 months [23,29,30] to nearly 2 years [24,39].

### 3.7. Functional Outcomes Associated with Weight-Bearing Protocols

In the isolated ORIF group, only six studies (43%, 6/14) explicitly mentioned pre-injury ambulatory functional status, typically describing patients as independently ambulatory without providing numeric scores [31,32,34,35,36,40]. Numeric quantification using a validated functional score was not reported in any isolated ORIF study. In contrast, in the combined ORIF and acute THA group, 13 studies (72%, 13/18) reported that patients were independently ambulatory prior to injury [26,27,28,29,30,32,34,35,36,38,39,40,41]. However, numeric quantification using a validated functional score was reported by only two studies: Hislop et al. [30] reported a median pre-injury Oxford Hip Score (OHS) of 44 points (range: 26–48), and Carrothers et al. [26] reported a median OHS of 43 points (range: 12–48).

Functional outcomes following isolated ORIF were generally moderate. Explicitly reported HHS ranged between 63 and 82 points [34,35,40,41], and OHS between 34 and 42 points [26,36]. SF-36 Physical Component Scores ranged from 36 to 47 points, reflecting moderate physical function similar to age-matched norms studies [25,31,35,41]. Independent ambulation rates after isolated ORIF varied notably (55% to 76%) [31].

In contrast, CHP procedures yielded HHS scores typically between 70 and 92, with multiple studies reporting excellent outcomes of 90 points or higher [23,34,39]. OHS ranged from 40 to 43 points, indicating consistently good outcomes [26,33,36]. SF-36 scores for combined ORIF and acute THA were better, particularly concerning lower bodily pain and higher physical function compared to isolated ORIF [25,35,36,41]. Furthermore, combined ORIF and acute THA demonstrated advantages regarding early postoperative mobilization, higher rates of independent ambulation, and superior patient satisfaction rates (74–90%) [28,29,31,32,35,38,39].

**Table 2:** Rehabilitation protocols, clinical outcomes, and functional results.

First Author	Rehabilitation Protocol (kg)	Duration of Partial Weight-Bearing (Weeks)	Time to Heal (Weeks)	LOS (Days)	Previous Functional Status	Functional Result (Follow Up)	Return to Preinjury Level
ORIF							
Boelch, 2016 [22]	PWB (20 kg)	6–12	NR	21	NR	At mean 12 months: bedridden (1), dependent on two crutches (1), ambulation with one crutch (2), independent walking without aids (6)	NR
Borg, 2009 [24]	PWB (NR)	8	12–24 (all healed by 6 months)	NR	NR	SF-36 (2 yrs): Physical function (67.5), Bodily pain significantly worse at 1-year vs. CHP group	NR
Carroll, 2010 [25]	NR	NR	12–24	NR	NR	MFA: 20.6; SF-36 Physical: 46.1; Mental: 51; SMFA bother: 25.7; SMFA dysfunction: 28.5	Similar outcomes to age-matched norms
Carrothers, 2024 [26]	PWB, TTWB (NR)	8	All healed by final follow-up (9 months)	16	Majority independently ambulatory without walking aids preinjury (median preinjury OHS 47)	OHS: 20 at 6 weeks, 33 at 6 months, 40.5 at 9 months	Independent walking without aids regained by 6 months
Jeffcoat, 2012 [31]	TTWB (NR)	8–12	12–24	12	NR	MFA: 15.8, SF-36 physical: 47.3, mental: 52.3, D’Aubigne mean: 16.2	Functional outcome similar to age-matched norms; 76% independent walking, remainder required cane or walker
Lannes, 2020 [32]	PWB (NR)	6–12	All fractures healed by 3 months	14	NR	HHS: 68.25	Good functional outcomes, independent walking in majority
Lont, 2019 [33]	PWB (NR)	6	All healed by 6 weeks	NR	Independently ambulatory pre-injury	OHS: 42	Patient satisfaction reported as generally good, with functional status similar to age-matched norms.
Manson, 2022 [34]	PWB, TTWB (NR)	8–12	NR	NR	Independent ambulatory before injury	HHS (12 months) 71.5; WOMAC 16.5, SF-36 PCS 45.3, SF-36 MCS 45.6	Similar functional outcomes compared to acute THA group
Navarre, 2020 [35]	PWB, TTWB (NR)	–12	All healed at follow-up (median 18 months)	NR	Independently ambulatory (no detailed score reported)	SF-12 PCS: 41.6; WOMAC: 83.9; HHS: 82.2; SMFA Function: 20.5; SMFA Bother: 17.6	Good functional outcomes, SF-12 PCS comparable to matched controls at 2 years; more than two-thirds working pre-injury returned to work within 1 year
Nicol, 2020 [36]	PWB (NR)	6–8	All healed initially	16	Independently ambulatory (majority ambulatory without aids, 9/14 independently ambulatory)	Functional status reported as good initially; OHS: 33.6	Majority regained independence; outcomes inferior to acute THA
O’Toole, 2014 [37]	TTWB (NR)	12	All healed initially by follow-up	NR	NR	WOMAC: 17, SF-8 PCS 46.1, SF-8 mental 54; 39% difficulty walking distances, 46% hip pain	Good functional outcomes; WOMAC and SF-8 scores similar or superior to matched norms; 39% difficulty walking long distances, 46% some hip pain, 17% taking medication for hip pain
Smakaj, 2022 [40]	PWB (NR)	6	All healed by follow-up	18	Independently ambulatory pre-injury	HHS (3 months): 66.3, SF-12 PCS (2 y): 40.6; SF-12 MCS 41.4	Moderate improvement in functional outcomes, no return to pre-injury level explicitly reported
Weaver, 2018 [41]	TTWB (NR)	6–12	All healed by final follow-up	NR	NR explicitly; independently ambulatory assumed pre-injury	HHS: 63; SF-36 Physical: 36; SF-36 Pain: 39	Functional limitations
ORIF/acute THA							
Boelch et al., 2016 [22]	PWB (20 kg), 2 patients immediate FWB	6 (3 pat), 12 (4 pat)	NR	25.6	NR	At mean 4.5 months: bedridden (1), dependent on two crutches (3)	NR
Boraiah, 2009 [23]	PWB, TTWB (NR)	TTWB 8 weeks, then PWB 4 weeks	12	NR	NR	HHS: 88, Excellent: 56%, Good: 25%, Fair: 18%	Ambulation restored in all patients except one (dementia)
Borg, 2009 [24]	FWB	0	12–24 weeks (all healed by 6 months)	NR	NR	SF-36 (2 yrs): Physical function 45, Bodily pain significantly better at 1 year vs. ORIF	NR
Carroll, 2010 [25]	NR	NR	NR	NR	NR	MFA 22, SF-36 Physical 57.9 (significantly better compared to ORIF group), SMFA bother 19.6, SMFA dysfunction 23.8	Comparable outcomes to age-matched norms
Carrothers, 2024 [26]	FWB	0	9 months	12	Majority independently ambulatory without walking aids preinjury (median preinjury OHS 43, range 12–48)	OHS: 27 at 6 weeks, 38 at 6 months, 43 at 9 months	Independent walking regained by 6 months; full return to preinjury residence by 12 weeks
Enocson, 2014 [27]	TTWB (NR)	12	12	NR	All independently ambulatory before fracture	HHS 88, SMFA dysfunction mean 29.6, SMFA bother 25.2, EQ-5D 0.65	All patients able to walk independently at final follow-up (48 months); Katz ADL: independent (9 pat), dependent in just one activity (2 pa)
Giunta, 2018 [28]	FWB	0	12–24	18	Majority independently ambulatory without walking aids, Devane score median 4	HHS: 70.4 (range 24–90), PMA: 14.3, EQ-5D mean: 0.65, SMFA dysfunction: 29.6, SMFA bother: 25.2	74% patients satisfied, 11% bedridden, 15% returned to skiing, Devane activity unchanged in 63% of patients
Herscovici, 2010 [29]	PWB (NR)	12	12–20 (mean 12 weeks)	8.1	Majority independent ambulators without aids preinjury	HHS: 74; Mean hip motion: Flexion 102°, Abduction 32°, Adduction 16°.	Walking without aids (7 patients), cane (5), walker (5), wheelchair-bound (1, dementia), majority able to return close to preinjury activity levels
Hislop, 2022 [30]	FWB	0	12–24	17.6	Majority independently ambulatory without walking aids, median pre-injury Oxford Hip Score 44 (26–48)	OHS 37.3 (28–48) at 1 year, EQ-5D mean 0.65, SMFA dysfunction mean 29.6, SMFA bother mean 25.2, Devane score 63% unchanged	60% mobilizing fully weight-bearing with walking aids by day 5 post-op
Lannes, 2020 [32]	FWB	0	All fractures healed by 3 months	11	NR (but independently ambulatory pre-fracture)	HHS 72.36	Good functional outcomes, independent walking (74% satisfied)
Lont, 2019 [33]	WBAT (NR)	0	All healed at 6 months	NR	Independently ambulatory pre-injury	OHS 41 (range 25–48) at 1 year; EQ-5D mean 0.65, SMFA Dysfunction mean 29.6, SMFA Bother mean 25.2	Slightly better pain scores and early mobilization advantages compared to ORIF alone, no significant difference in overall functional scores at longer-term follow-up
Manson, 2022 [34]	PWB, TTWB (NR)	12	All healed at follow-up	NR	Independent ambulatory pre-injury status required	HHS 92 (1-year), WOMAC 16.5, SF-36 PCS 45.3, SF-36 MCS 45.6 (no significant clinical differences between groups except HHS favored acute THA group, *p* = 0.07)	Similar satisfaction and function outcomes to ORIF alone
Navarre, 2020 [35]	FWB	0	All healed by follow-up	NR	Independently ambulatory pre-injury	Functional scores similar to ORIF alone; no significant differences explicitly reported	No major differences
Nicol, 2020 [36]	FWB	0	All healed postoperatively	15	Independently ambulatory pre-injury (10/12 independently ambulatory pre-injury)	OHS: 40.1	Better outcomes than delayed THA; most regained independent mobility
Rickman, 2014 [38]	FWB	0	12–24	18	Independently ambulatory pre-injury	All patients mobilized fully WB by day 7 postoperatively; independent ambulation (with minimal aids at most) achieved by 6 months	All patients returned to preinjury residential status by 12 weeks
Salama, 2016 [39]	PWB, TTWB (NR)	6	All fractures healed by follow-up (mean 21.7 months)	NR	Independently ambulatory pre-injury (explicit scoring NR)	HHS: 90.4 (final follow-up), 72.2% excellent, 27.8% good outcomes.	All patients regained independence at final follow-up
Smakaj, 2022 [40]	FWB	0	All healed by final follow-up	18	Independently ambulatory pre-injury	HHS: 73.6, SF-12 PCS (2 y): 41.5; SF-12 MCS: 42.8	Good to excellent outcomes, functional independence achieved by all at final follow-up, overall better functional outcomes vs ORIF group
Weaver, 2018 [41]	FWB in 48% patients, 52% WBAT	0	All healed by follow-up	NR	Independently ambulatory pre-injury	HHS: 82, SF-36 pain: 48, physical: 41	Good outcomes: significantly better functional/pain scores than ORIF-alone, improved function and less pain

NR = not reported—studies did not specify weight-bearing recommendations. PWB = partial weight-bearing, FWB = full weight-bearing, TTWB = toe-touch weight-bearing, WBAT = weight bearing as tolerated. HHS = Harris Hip Score, SF-36 PCS, MCS, MFA = Musculoskeletal Functional Assessment, OHS = Oxford Hip Score, EQ-5D, SMFA = Short Musculoskeletal Functional Assessment, PMA = Postel-Merle Aubigné, Katz ADL.

### 3.8. Complications, Reoperation and Mortality Rates

#### 3.8.1. General Complications

General complications were reported clearly in 12 [22,24,25,26,28,29,30,31,32,33,39,40] out of 20 studies (60%) (Table 3). For isolated ORIF, complication rates ranged broadly from 17% to 59% and predominantly included superficial infections (up to 13% [40]), hematoma formation (17% [22]), and significant intraoperative bleeding (5% [26]). In the CHP group, general complications were reported in 14 studies (78%), ranging from 6% [23] to 74% [28], including superficial infections (up to 14% [40]), surgical site infections (15% [28]), wound healing disorders (up to 11% [28]), and pressure sores (7% [28]). Importantly, the term “significant intraoperative bleeding” (5%) in the isolated ORIF group, as reported by Carrothers et al. [26], corresponded to an average intraoperative blood loss of approximately 550 mL (range: 93–3000 mL) and a transfusion rate of around 33%. In contrast, anemia documented in the CHP group by Giunta et al. [28] was notably higher, affecting 63% of patients. The CHP group generally exhibited higher blood loss, averaging 827 mL (range: 152–3019 mL) according to Giunta et al. [28] and 1163 mL (range: 300–4500 mL) as reported by Herscovici et al. [29], with transfusion rates reported between approximately 37% and 46% [28,32]. None of the reviewed studies explicitly reported 30-day readmission rates.

#### 3.8.2. Systemic Complications

Systemic complications were well documented in 10 studies (50%) [22,25,26,28,29,30,31,36,38,41]. Following isolated ORIF, reported systemic complications included pulmonary embolism and deep vein thrombosis (1–2% [25,31]), pneumonia (up to 2% [31]), perioperative myocardial infarction [41], and death from renal insufficiency (4% [22]). In the CHP group, systemic complications were explicitly higher, notably anemia (up to 63% [28]), cardiac distress (up to 11% [28,30]), pulmonary embolism (up to 11% [28,30]), urinary tract infections (9% [29,30]), deep vein thrombosis (up to 7% [30]), transient ischemic attack (TIA; 5% [29,30]), and acute kidney injury (10% [30]).

#### 3.8.3. Orthopedic Complications

Orthopedic complications were thoroughly described in 16 studies (80%) [22,24,26,27,28,29,30,31,32,34,35,36,38,39,40,41]. In the ORIF-alone group, the most frequent complication was severe post-traumatic arthritis frequently necessitating conversion to delayed THA. Additionally, heterotopic ossification occurred frequently (up to 24%, [32]; 21%, [40]), avascular necrosis (up to 12% [31]), and intra-articular hardware placement (up to 10% [31]).

In the CHP group, heterotopic ossification was predominant, reported in up to 62% [24], though mostly mild. Dislocations occurred in approximately 8–11% [29,30,41], acetabular component loosening or migration in up to 18% [29], and instability requiring surgical intervention in 5–10% [26,41].

After isolated ORIF, neurological complications included lateral femoral cutaneous nerve palsy (31–34% [25,31]) and postoperative foot drop (2.4% [31]). In the CHP group, neurological complication rates were explicitly reported to be around 7% [27,28]; however, specific types of nerve injuries were less frequently detailed.

**Table 3:** Complications and reoperation rates.

First Author	General Complications	Systemic Complications	Orthopedic Complications	Reoperations
ORIF				
Boelch, 2016 [22]	Hematoma (17.4%), superficial infection (8.7%)	Death from renal insufficiency (4.3%)	Nerve injury (4.3%), AVN (4.3%), infectious necrosis (4.3%), femoral head fracture (4.3%), central protrusion (4.3%), intra-articular screw placement (4.3%)	Total reoperations: 43.5% (THA 9, Girdlestone 1)
Borg, 2009 [24]	NR	NR	Osteoarthritis (14.3%), femoral head osteonecrosis (7.1%), hip dislocation (7.1%), HO (14.3%)	Total reoperations: 71.4% (9 conversions to THA, 1 Girdlestone)
Carroll, 2010 [25]	Superficial infection (3.23%)	Pulmonary embolism (1.08%), DVT (1.08%), pneumonia/respiratory failure death (1.08%)	Intra-articular hardware (3.23%), foot drop (1.08%), hernia (1.92%), lateral femoral cutaneous nerve palsy (31%), loss of reduction (6.45%),	Total reoperations: 30.95% (26 conversions THA, 1 Girdlestone)
Carrothers, 2024 [26]	Significant intraoperative bleeding (5%)	NR	Femoral head fracture (5%), severe osteoarthritis (5%)	Reoperation for femoral head fracture (5%)
Jeffcoat, 2012 [31]	Superficial infection (4.9%)	Pulmonary embolism (2.4%), DVT (2.4%), femoral artery thrombosis (2.4%), pneumonia (2.4%)	HO Brooker I–II (9.8%), avascular necrosis (12.2%), arthritis (26.8%), intra-articular hardware (9.8%), foot drop (2.4%), hernia (4.9%), lateral femoral cutaneous nerve palsy (31%)	Reoperations (hardware removal 9.8%, embolectomy 2.4%)
Lannes, 2020 [32]	Total 32%; superficial infection (4%)	NR	Osteoarthritis requiring THA (16%), avascular necrosis, intra-articular hardware removal (3.23%), HO (23.8%), nerve palsy (8%)	Total reoperations: 20%; secondary THA (16%), surgical site infection requiring reoperation (4%)
Lont, 2019 [33]	Deep infection (8%)	NR	Osteoarthritis requiring THA (36%), hip dislocation (3%), periprosthetic femur fracture (3%)	Total reoperations: 36% (secondary THA)
Manson, 2022 [34]	Total: 58.5%; infection (4.9%)	Pneumonia-related death (2.4%)	Posttraumatic arthritis (41%), HO (9.8%), femoral nerve palsy (2.4%), deep infection (4%), nerve palsy (2.4%)	Total reoperations: 45% (conversion THA 41%, infections, HO, hardware removal (9.8%))
Navarre, 2020 [35]	NR	NR	Severe osteoarthritis requiring THA (23.6%), infections and nerve palsies (not explicitly quantified separately)	Conversion to THA: 23.6%
Nicol, 2020 [36]	NR	NR	Severe post-traumatic arthritis (16.5%)	Conversion to THA: 16.5%
O’Toole, 2014 [37]	NR	NR	Post-traumatic arthritis (28%), hip pain (46%), walking difficulty (39%), minor complications (nerve palsies or HO NR)	Conversion to THA: 28%
Smakaj, 2022 [40]	Total complications: 41.6%; wound infection (12.5%)	Deep vein thrombosis (DVT, 8.3%)	Severe arthritis (29.2%), HO (20.8%)	Conversion to THA: 29%
Weaver, 2018 [41]	Total complications: 30%	Perioperative myocardial infarction in 1 patient	Severe post-traumatic arthritis, severe HO (9.1%); infection requiring operative debridement (12%)	Total reoperation rate: 30%; conversion THA 21%, infection requiring surgery (12.1%), HO excision (3%)
ORIF/acute THA				
Boelch et al., 2016 [22]	NR	NR	Subluxation (11.1%), acetabular dislocation (11.1%), loosening (11.1%), HO Brooker III (11.1%)	Revision surgeries: 22%
Boraiah, 2009 [23]	Superficial infection (5.5%)	NR	HO Brooker III, bilateral paresthesia (5.5%), recurrent dislocation (5.5%), femoral stem loosening (5.5%)	Revision for dislocation (5.5%)
Borg, 2009 [24]	NR	NR	No dislocations or implant loosening (0%), HO (Grade I: 3 patients, Grade II: 1 patient, Grade III: 4 patients)	0%
Carroll, 2010 [25]	NR	NR	Dislocation (2.86%), infection requiring revision (3.2%)	NR
Carrothers, 2024 [26]	Superficial infection (4.8%)	Pulmonary embolism preop (4.8%)	Early hip dislocation requiring reduction (10%)	Early dislocation reduced (9.5%), superficial infection debrided (4.8%)
Enocson, 2014 [27]	Superficial wound infection (5.5%), bilateral paresthesia (5.5%)	DVT (5.5%)	Recurrent hip dislocation (5.5%), femoral stem loosening (5.5%), HO (Brooker Grade I–III) (4 patients total: Grade I: 2 patients, Grade II: 1 patient, Grade III: 1 patient)	5.5% (1 revision due to recurrent dislocation)
Giunta, 2018 [28]	Surgical site infection (15%), pressure sores (7.4%), cicatrization disorder (11%)	DVT (7.4%), pulmonary embolism (11%), cardiac distress (11%), anemia (63%)	HO (Grade I–III: total 4 patients), early hip dislocation (11%), nerve injuries (7.4%)	NR
Herscovici, 2010 [29]	Superficial wound infection (4%), wound dehiscence (4%)	UTI (9%), TIA	HO (18.2%), recurrent hip dislocations (23%), osteolysis and component loosening (18%)	Reoperations: 22.7% (component revision, recurrent dislocation)
Hislop, 2022 [30]	Total complications 59%; significant: urinary tract infections (9%), transient ischemic attack (4.5%)	Acute kidney injury (10.5%), hospital-acquired pneumonia (10.5%), iliac artery embolism (1.8%), DVT (7.4%), pulmonary embolism (11%), cardiac distress (11%), anemia (63%)	Hip dislocations (8.8%, 5 patients; 4 treated closed, 1 excision arthroplasty), prosthetic joint infection (5.3%), acetabular component migration requiring revision (1.8%), femoral head fracture requiring THA (1.8%), severe osteoarthritis (1.8%), HO Brooker III (1.8%)	Reoperations total: 8.8%; revision due to recurrent dislocation (2), component loosening requiring revision (4), superficial infection debridement (1)
Lannes, 2020 [32]	Total 31%; Surgical site infection (7.7%)	DVT (6.7%)	Dislocations (7.7%), neuralgia (6.7%), HO Brooker III (6.7%), infection (7.7%)	Surgical site infection requiring reoperation (7.7%)
Lont, 2019 [33]	NR	NR	Hip dislocation (3%), periprosthetic femur fracture (3%), no infections	Total reoperations: 6% (revision arthroplasty due to dislocation 3%, periprosthetic fracture 3%)
Manson, 2022 [34]	Total: 8%	NR	Superficial wound dehiscence without infection requiring closure (4%), 1 patient procedure aborted due to instability (4%)	Total reoperations: 8% (2 patients, superficial wound debridement and closure)
Navarre, 2020 [35]	High early mortality, other complications NR	NR	NR	NR
Nicol, 2020 [36]	NR	NR	Dislocation (8.3%), infection (8.3%)	Reoperations: 8.3% (infection, subsequent instability)
Rickman, 2014 [38]	Total complications 8.3%: superficial infection, perioperative mortality (4.2%)	Myocardial infarction (4.2%), DVT (4.2%)	No dislocations or implant loosening (0%)	Reoperations: 4.2% (1 patient due to superficial infection)
Salama, 2016 [39]	Total complications: 11% heterotopic ossification (grade I and III, no functional consequences), 5.6% medial acetabular cup migration (2 mm, asymptomatic), no dislocations, infections or delayed union	NR	HO (Brooker I and III, 11%), medial acetabular migration (asymptomatic, 5.6%), no loosening or dislocations	0
Smakaj, 2022 [40]	Total complications: 42.9%; wound infection (14.3%)	DVT, 4.2%	Implant migration (5.6%), HO (14.3%), no loosening, no dislocations, no explicit deep infections; secondary osteoarthritis not explicitly stated	0
Weaver, 2018 [41]	Total complications: 14%;	NR	Dislocations (11%), instability requiring revision (5%), infection requiring debridement (8.1%), severe heterotopic ossification (11%), no component loosening or migration	Total reoperation rate: 14%; infection requiring debridement (8.1%), recurrent instability requiring revision (5%)

NR = not reported, AVN = avascular necrosis, THA = total hip arthroplasty, DVT = deep vein thrombosis, UTI = urinary tract infection, TIA = transient ischemic attack, HO = heterotopic ossification.

#### 3.8.4. Reoperations and Conversions to Delayed THA

**Figure 4:**
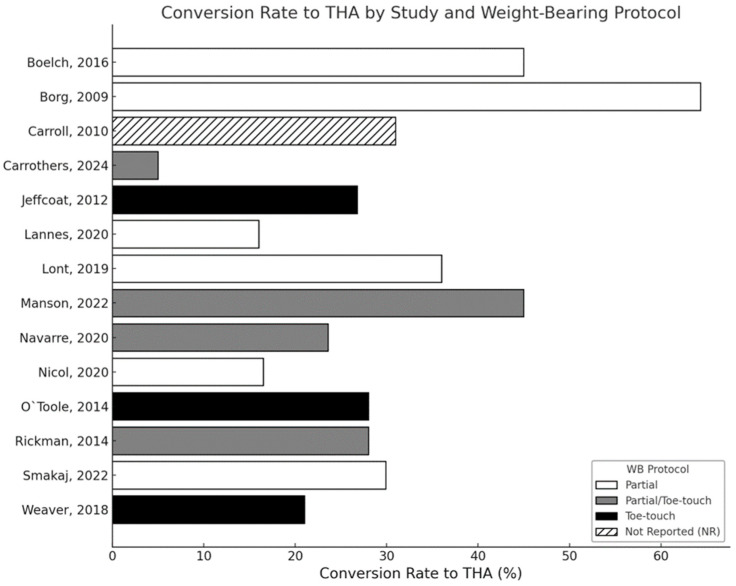
Conversion rates to total hip arthroplasty (THA) following operative treatment of acetabular fractures, sorted by study and postoperative weight-bearing (WB) protocol. None of the included studies utilized a full weight-bearing protocol. “NR” (not reported) indicates that the study did not specify the postoperative weight-bearing protocol [22,24,25,26,31,32,33,34,35,36,37,38,40,41].

Reoperation rates were well documented in 16 studies (80%) [22,25,26,29,30,31,32,33,34,35,36,37,38,39,40,41]. In the isolated ORIF group, reoperations were predominantly driven by conversion to delayed THA due to severe post-traumatic arthritis or avascular necrosis, with conversion rates reported between 17% and 45% (45% [34], 36% [33], 31% [25], 29% [40], 28% [38], 28% [37], 24% [35], 21% [41], 17% [36]) (Figure 4). As most outcomes were reported heterogeneously across studies (e.g., HHS, complication types, LOS), a formal I^2^ and τ^2^ analysis was only feasible for delayed THA conversion rates following ORIF. This exploratory random-effects analysis revealed substantial heterogeneity (I^2^ = 84.7%; τ^2^ = 0.018), indicating considerable variation in reported conversion rates across studies. Other reasons for reoperations in the isolated ORIF group included infection requiring surgical debridement (12% [41]), hardware removal (10% [31], 10% [34]), and excision of heterotopic ossification (3% [41], 4% [34]). In the combined ORIF and acute THA group, total reoperation rates were generally lower, explicitly ranging from 4.2% [38] to 23% [29]. Common reasons included recurrent dislocations (10% [26], 11% [41]), component loosening (18% [29]), and infections necessitating operative debridement or revision (8% [41], 8% [36], 5% [26]).

#### 3.8.5. Mortality

Mortality rates were documented clearly in 14 of the 20 reviewed studies (70%) and included early (in-hospital and 30-day), intermediate (1-year), and long-term (3–5 years) outcomes [22,24,25,26,29,30,33,34,35,36,37,38,40,41]. Early mortality was generally low in both treatment groups. In the isolated ORIF group, early mortality rates ranged between 0% and 3.2%, with the majority of studies reporting 0% [24,26,34]. In the combined ORIF and acute THA group, early mortality ranged from 0% to 7%, with most studies likewise reporting rates close to zero [24,29,34]. At one-year follow-up, mortality rates were highly variable. For isolated ORIF, reported mortality rates varied widely from 0% to as high as 25%, commonly around 12–15% [37]. For the combined ORIF and acute THA group, one-year mortality also showed considerable variability, ranging from 0% to 15%, with many studies similarly reporting intermediate values around 12–15% [30]. Long-term mortality at 3 to 5 years was substantially higher in both groups. In the isolated ORIF group, explicitly documented long-term mortality reached up to 42% at five years [33]. In the combined ORIF and acute THA group, reported long-term mortality was similarly high, ranging from 23% at approximately two to four years [24] to as high as approximately 70% at five years [33].

## 4. Discussions

This systematic review was performed to shed light on the uncertainties and variability in postoperative rehabilitation protocols for older adults after the surgical management of acetabular fractures, particularly regarding weight-bearing restrictions. We aimed to investigate whether different WB strategies are utilized after ORIF or after CHP, and whether or not these correlate with distinct clinical outcomes.

A key finding of this review is the striking variability in WB protocols. Although 95% of the studies reported postoperative WB recommendations, no consensus is evident about the optimal practice. To directly address the first research question— Do reported rehabilitation protocols differ according to the type and timing of postoperative weight-bearing (WB) restrictions?—our findings demonstrate a clear and consistent pattern: WB protocols vary substantially depending on surgical strategy. ORIF was uniformly associated with restricted WB, whilst CHP patients were usually allowed early full WB. This reflects distinct biomechanical requirements: isolated ORIF demands stable fixation and biological healing to prevent secondary displacement under load, whereas CHP immediately restores joint stability through prosthetic replacement, theoretically facilitating early weight-bearing despite ongoing fracture healing [43]. However, the marked variability in reported WB protocols highlights the lack of standardized, evidence-based rehabilitation pathways for older adults with acetabular fractures. Although most studies reported WB protocols, specific definitions, loading thresholds, and durations of restriction varied widely and often lacked clear justification.

Interestingly, while non-weight-bearing (NWB) or strict weight-bearing limitations are frequently prescribed after ORIF of acetabular fractures, studies from proximal femoral fractures indicate that strict NWB is practically challenging, if not impossible, for older patients to adhere to [44,45]. In fact, recent evidence demonstrates that most patients substantially exceed prescribed partial weight-bearing limits without immediate radiographic signs of secondary fracture displacement at early follow-up, although long-term effects on fixation failure or displacement remain uncertain [46]. In the context of acetabular fractures, the evidence supporting early full weight-bearing is limited. A review by Murena et al. [17] indicates that, while early weight-bearing may stimulate fracture healing and functional recovery, there is a lack of robust clinical evidence to support this approach universally. Similarly, Giordano et al. [5] conducted a systematic review and meta-analysis on postoperative rehabilitation protocols for acetabular fractures, concluding that early permissive weight-bearing does not appear to increase the risk of loss of reduction or complications. However, the patient population in this review primarily comprised younger adults (mean age 35 years), which limits applicability of these findings to older patients.

Addressing the second research question—Do surgeons typically restrict weight-bearing after ORIF to protect fixation integrity?—our review confirms that this practice is consistently applied. Across all ORIF studies, restricted WB was recommended for several weeks, based on the assumption that early loading might jeopardize fixation in osteoporotic bone. Importantly, the same biomechanical concerns used to justify WB restriction in ORIF—such as the need for biological integration in compromised bone—also apply to components of CHP, including cages, uncemented sockets, and bone grafts. Yet, full WB was often permitted in these cases. This discrepancy highlights the need to clearly differentiate between reconstruction of the joint surface through prosthetic replacement, which may allow immediate loading, and ORIF procedures addressing intra-articular fractures, which typically demand anatomical reduction, biological healing, and consequently restricted WB [17].

However, this rationale remains largely theoretical. None of the included studies used objective tools—such as in-shoe pressure sensors [46,47]—to monitor patient adherence or assess whether or not WB restrictions reduced complication rates. The protective effect of delayed WB, therefore, remains unproven. This represents a significant methodological gap, as it remains unknown whether patients complied with prescribed protocols or whether non-adherence affected outcomes. Wearable sensor technologies, such as in-shoe pressure sensors, have been successfully employed to objectively monitor adherence to postoperative weight-bearing protocols. Braun et al. utilized these sensors specifically after acetabular fracture fixation, demonstrating low adherence without negative short-term effects on fixation [45]. Additionally, similar wearable sensors have effectively quantified and improved compliance through biofeedback mechanisms in lower extremity fractures [46,47]. However, the systematic use of these technologies in acetabular fracture rehabilitation remains limited, representing a critical area for future research.

Addressing the third research question—Is the decision to perform a “fix-and-replace” procedure primarily driven by the aim of permitting immediate full WB postoperatively, or is it predominantly based on established fracture-specific indications?—our findings indicate that immediate postoperative WB capability alone was rarely the primary indication for selecting CHP. Instead, surgical decisions predominantly relied on established fracture-specific criteria, such as severe joint comminution, femoral head damage, substantial acetabular impaction, or symptomatic pre-existing osteoarthritis. Although early postoperative mobilization and full weight-bearing capability were occasionally cited as additional advantages, these did not constitute stand-alone indications. To date, in the absence of clear evidence demonstrating the safety, efficacy, and superiority of unrestricted WB after CHP [48], there is currently no rationale for choosing CHP primarily as a means to facilitate immediate postoperative WB. This distinction is clinically important, as it underscores that early WB has not yet been validated as an independent indication for acute THA. Future trials should specifically assess whether or not such a strategy may be justified in selected borderline cases.

Patient selection clearly emerged as another key determinant of treatment choice. Patients receiving CHP tended to have slightly higher mean ages and were more frequently reported to have higher comorbidity indices, more complex fracture morphologies, and a greater prevalence of cognitive impairment. Although the numerical difference in mean age (approximately four years) might be clinically minor, CHP procedures were typically chosen for cases involving more challenging clinical conditions or complex fractures. Such selection bias complicates direct outcome comparisons and likely contributes to the higher complication rates observed in the CHP group.

Additionally, our findings also suggest a gradual shift in clinical practice toward the broader use of CHP in older patients with osteoporotic or comminuted fractures [32]—likely reflecting improvements in implant technology, surgical techniques, and surgeon confidence. While promising, this development requires critical evaluation of long-term outcomes and patient-centered benefit–risk balances.

The fourth research question—Is unrestricted postoperative weight-bearing correlated with shorter hospitalization times, faster healing, superior functional outcomes, lower systemic complications, and decreased mortality, or does restricted postoperative weight-bearing result in fewer orthopedic complications and lower reoperation rates?—yielded nuanced results. CHP with immediate full WB was associated with earlier mobilization, shorter hospital stays, improved functional scores (e.g., higher Harris Hip Scores), and greater patient satisfaction. However, these benefits were accompanied by a higher rate of systemic complications, including anemia (up to 63%), pulmonary embolism (up to 11%), deep vein thrombosis (up to 7%), and pressure sores. In comparison, thromboembolic events were reported in only 1–2% of patients following ORIF. This apparent contradiction indicates that permitting immediate full WB after CHP does not necessarily translate into actual early mobilization, as objective measures of patient compliance or capability were rarely employed. Indeed, Corcoran et al. [49] emphasized that early permission for mobilization alone is insufficient, since inactivity and bedrest independently increase complication risks (“use it or lose it”). Consequently, the higher complication rates observed in the CHP group might reflect inherently more invasive surgery and differences in patient selection, with CHP patients generally being older and more comorbid than those treated with ORIF. Such factors likely limited actual mobilization despite permission for immediate WB. Conversely, prolonged WB restrictions after ORIF may also contribute to immobility-related morbidity, such as muscle atrophy, pneumonia, and impaired functional recovery—particularly in older, cognitively impaired, or otherwise vulnerable patients [17,41]. Thus, when deciding between unrestricted and restricted WB protocols, surgeons must carefully balance mechanical protection against risks related to prolonged immobilization. An important additional finding regarding mortality was that long-term mortality rates (3–5 years) appeared comparable between the ORIF and CHP groups, indicating that, from a survival perspective, the surgical method alone does not decisively impact long-term mortality. Isolated ORIF is indeed associated with lower rates of dislocation and implant loosening, as expected in procedures without prosthetic components. However, this benefit came at the cost of higher delayed THA conversion rates (17–45%) due to post-traumatic arthritis, avascular necrosis, or functional deterioration over time.

This study has several limitations, predominantly related to the nature and quality of the existing evidence. Most included studies were observational with moderate to high risk of bias, inherently limiting the strength of our conclusions. WB protocols across studies were inconsistently defined and infrequently accompanied by objective outcome metrics or compliance data. Specifically, terms such as “partial” or “toe-touch” WB often lacked standardized definitions or quantification, severely limiting interpretability and direct comparability between studies. Moreover, the majority of studies did not objectively verify whether or not patients adhered to prescribed WB restrictions, representing a critical methodological flaw. To mitigate these limitations, we performed a systematic and rigorous narrative synthesis rather than a quantitative meta-analysis, clearly highlighting heterogeneity in rehabilitation protocols and study designs. We transparently reported these methodological issues, explicitly noting the gaps in evidence and emphasizing areas that urgently require higher-quality research. While these limitations restrict definitive clinical recommendations, our review distinctly outlines the current state of evidence and underscores critical research priorities for future studies.

The strength of this systematic review lies in addressing a clinically important yet previously underexplored topic—postoperative rehabilitation, specifically weight-bearing protocols, following acetabular fracture surgery in older adults. To our knowledge, this is the first systematic review explicitly comparing ORIF and CHP in this context, thereby providing new and clinically relevant insights. We applied a rigorous and transparent methodology, adhering closely to PRISMA guidelines, to systematically identify, select, and narratively synthesize evidence from 20 relevant studies. By highlighting consistent trends, outcome differences, and substantial knowledge gaps, our findings offer a robust foundation that clearly defines areas requiring future investigation. Consequently, our results have the potential to directly inform and drive subsequent research, ultimately guiding the development of standardized and evidence-based rehabilitation protocols.

## 5. Conclusions

In summary, considerable variability and uncertainty persist in rehabilitation strategies for older adults with acetabular fractures. While early full WB after CHP may promote faster recovery, it is associated with increased systemic risks. In contrast, delayed WB after ORIF may reduce orthopedic complications but carries its own burden of immobility-related morbidity. As emphasized, clinical decisions must carefully balance the benefits and risks of different weight-bearing strategies. This review strongly advocates for an evidence-based shift towards individualized rehabilitation strategies, structured mobilization programs, and rigorous adherence monitoring through objective measurement tools (e.g., wearable sensors). Future prospective trials must evaluate fracture-specific and patient-centered criteria to optimize rehabilitation strategies and clarify the true balance between mechanical protection and functional recovery in this vulnerable population. For an individualized postoperative rehabilitation protocol after surgical management of acetabular fractures in older adults, factors such as fracture pattern (stable vs. unstable), bone quality (normal vs. osteoporotic), fixation strength (stable vs. unstable), and the patient’s ability (e.g., motivation, cognition, obesity) to comply with partial weight-bearing limitations should be carefully considered. Furthermore, decisions regarding ORIF versus CHP should be based on established indications, including pre-existing symptomatic osteoarthritis, femoral head injury, the ability to anatomically reconstruct the articular surface within a reasonable time frame, and the patient’s overall medical fitness. Ultimately, interdisciplinary and interprofessional collaborative research efforts are required to strengthen the evidence base, guide standardized rehabilitation protocols, and enhance patient-centered outcomes.

## Figures and Tables

**Table 1:** Overview of included studies and patient characteristics.

First Author	Study Design (Level of Evidence)	Mean Age (Years)	Number of Patients	Classification of Fractures	Treatment	Approach	Mean Follow Up (Months)
Boelch, 2016 [22]	Retrospective clinical study (4)	1. 73.4, 2. 79.8	32	1. BC, PW, AC, PW/PC, ACPHT 2. AC, Tr, AW, BC	1. ORIF 2. ORIF + acute THA (anti-protrusion cage +/− posterior column bridge plating)	1. KL, modified ilioinguinal (Letournel), KL + Stoppa, comb 2. KL, Bauer	1. 9.5, 2. 4.5
Boraiah, 2009 [23]	Retrospective case series (4)	72	18	PW, Tr, ACPHT, BC, Femoral head impaction	ORIF + acute THA	KL	46.8
Borg, 2009 [24]	Prospective comparative study (2b)	1. 68.2, 2. 76.5	27	1. PW, ACPHT, BC 2. PW, PC/PW, Tr/PW, ACPHT, BC	1. ORIF2. ORIF + acute THA with titanium Burch–Schneider cage	1. KL, ilioinguinal, comb. 2. KL	≥24
Carroll, 2010 [25]	Retrospective case series from prospectively maintained database (4)	67	93	BC, ACPHT, PW, T, AC, PC/PW, AW, Tr	1. ORIF 2. ORIF + acute THA	1. Ilioinguinal, extended iliofemoral 2. NR	63
Carrothers, 2024 [26]	Prospective randomized controlled feasibility trial (1b)	1. 71.7, 2. 79.2	60	ACPHT, BC, AC, PC	1. ORIF 2. ORIF + acute THA	NR	Median: 9
Enocson, 2014 [27]	Prospective cohort study (2b)	75.5	15	AC, ACPHT, Tr	ORIF + acute THA (Burch–Schneider reinforcement ring)	Hardinge, Moore	46.8
Giunta, 2018 [28]	Retrospective cohort study (2b)	68.5	27	PW, AC, PC, T, ACPHT	ORIF + acute THA (acetabular reinforcement cross-plate)	KL, Moore, anterior	48
Herscovici, 2010 [29]	Retrospective study (4)	75.3	22	Tr, PW, ACPHT, BC, femoral head involvement, hip dislocation	ORIF + acute THA (Ganz ring acetabular component or Osteonics THA components)	KL, ilioinguinal	29.4
Hislop, 2022 [30]	Retrospective review of prospectively collected data (2b)	77	57	ACPHT, BC, PC, PC/PW, Tr	ORIF + acute THA (Trabecular Metal Acetabulum Revision System (TMARS))	Stoppa ± ilioinguinal, KL	35.5
Jeffcoat, 2012 [31]	Retrospective cohort comparison analysis (2b)	67	41	ACPHT, BC, AC, T	ORIF	Ilioinguinal (limited or full)	63
Lannes, 2020 [32]	Retrospective cohort study (2b)	1. 75, 2. 78	51	1. PW, PC, AW, Tr, ACPHT, BC 2. Tr, ACPHT, BC, T	1. ORIF 2. ORIF + acute THA (Ganz ring)	1. Anterior, KL, comb. 2. Anterior, anterolateral, posterior	Median: 12
Lont, 2019 [33]	Retrospective cohort study (2b)	1. 69, 2. 71	59	1. PW, AW, AC, Tr, ACPHT, BC 2. PW, Tr, ACPHT, T, BC	1. ORIF 2. ORIF + acute THA (reinforcement ring)	1. Anterior intrapelvic, ± lateral window (ilioinguinal), KL 2. Direct later, posterolateral	48 (minimum 24 months)
Manson, 2022 [34]	Prospective comparative clinical trial (2b)	1. 70.7, 2. 72.8	47	1. Dome impaction, PW, femoral head fracture 2. Dome impaction, PW, femoral head fracture	1. ORIF 2. ORIF + acute THA	1. KL, Stoppa 2. KL, Stoppa	12
Navarre, 2020 [35]	Retrospective registry-based observational study (2b)	1. 69.7, 2. 78	80	1. AC, PW, Tr, PC/PW, ACPHT, BC 2. PW, Tr, BC	1. ORIF 2. ORIF + acute THA	1. KL, Stoppa, ilioinguinal, comb 2. KL	Median 18
Nicol, 2020 [36]	Retrospective cohort study (2b)	1. 76, 2. 81	26	1. AC, BC, Tr, T, PC/PW 2. ACPHT, BC, T, PC/PW, AC	1. ORIF 2. ORIF + acute THA (TMARS or similar)	1. Ilioinguinal, Stoppa, KL, SHD 2. KL	60
O’Toole, 2014 [37]	Retrospective review (4)	69.7	46	PW, PX/PC, Tr, ACPHT, BC, other	ORIF	1. KL, ilioinguinal, Stoppa, comb.	53
Rickman, 2014 [38]	Retrospective cohort study (2b)	2. 77	24	AC, BC, Tr, PW, PC	ORIF + acute THA (Burch–Schneider cage)	Stoppa + KL	24
Salama, 2016 [39]	Retrospective cohort study (2b)	66.1	18	PW, Tr, ACPHT, BC, T, significant femoral head involvement in some cases	ORIF + acute THA	KL, ilioinguinal + posterior	21.7
Smakaj, 2022 [40]	Retrospective multicentric cohort study (2b)	1. 69.5, 2. 73.4	45	1. ACPHT, BC, PW/PC, T, Tr, PW, AC, PC 2. ACPHT, BC, PW/PC, T, Tr, PW, AC, PC	1. ORIF 2. ORIF + acute THA (Burch–Schneider cage)	1. KL, Stoppa, comb. 2. KL, comb.	Min 24
Weaver, 2018 [41]	Retrospective cohort study (2b)	1. 73, 2. 79	70	1. PW, PC, PW, ACPHT, BC, other 2. AC, PW, ACPHT, BC, Tr/PC, T, other	1. ORI 2. ORIF + acute THA	1. KL, ilioinguinal 2. NR	1. 22, 2. 18

AW = anterior wall, AC = anterior column, ACPHT = anterior column posterior hemitransverse, PW = posterior wall, PC = posterior column, BC = both columns, Tr/PW = transverse posterior wall, T = T-type, PC/PW = posterior column/posterior wall, Tr = transverse, ORIF = open reduction and internal fixation, THA = total hip arthroplasty, KL = Kocher–Langenbeck approach, SHD = surgical hip dislocation, comb = combined approaches.

## Data Availability

The data presented in this study are available on request from the corresponding author.

## Supplementary Materials

The following supporting information can be downloaded at: https://www.mdpi.com/article/10.3390/jcm14144912/s1. Table S1: Search strategy.

## Abbreviations

The following abbreviations are used in this manuscript:

ORIF	open reduction and internal fixation
THA	total hip arthroplasty
CHP	combined hip procedure
WB	weight-bearing
NWB	non-weight-bearing
LOS	length of stay
ACPHT	anterior column posterior hemitransverse
ASA	American Society of Anesthesiologists
CCI	Charlson Comorbidity Index
OHS	Oxford Hip Score
