# Supplementary material for: Rehabilitation Protocols for Surgically Treated Acetabular Fractures in Older Adults: Current Practices and Outcomes
Source: J Clin Med. 2025 Jul 10;14(14):4912. doi: 10.3390/jcm14144912 (PMC12294826; doi:10.3390/jcm14144912)
Supplement: Supplementary file 1 [file jcm-14-04912-s001.zip › jcm-3716101-supplementary.pdf]

Supplementary Materials

S1. Search Methods for Identification of Studies

The search scope was broadened using the "*" symbol, for example, "Acetabul*" was used to include both acetabulum and acetabular terms. The search was expanded using the "OR" operator, while the "AND" operator was used to refine it. Table S1 provides a comprehensive list of all the XX synonyms and variations included in the search strategy.

Table S1. Search strategy

Term	Synonym searched
**Acetabul***	Hip OR Pelv*
	AND
**Fractur***	injur* OR "periprosthetic fracture*
	AND
**Geriatric**	elder* OR senior OR older
	AND
**Operat***	postoperat* OR surg*
	AND
**Limit***	limit* OR treatment OR therapy OR aftercare OR rehabilitation OR physio* OR restrictions OR recommendations OR "weight bearing" OR strategy OR management
	NOT
**Conserv***	"non-surgical" OR "non-invasive" OR "non-operative
